# Sleep, Little Baby: The Calming Effects of Prenatal Speech Exposure on Newborns’ Sleep and Heartrate

**DOI:** 10.3390/brainsci10080511

**Published:** 2020-08-02

**Authors:** Adelheid Lang, Renata del Giudice, Manuel Schabus

**Affiliations:** 1 Department of Psychology, Centre for Cognitive Neuroscience (CCNS), University of Salzburg, 5020 Salzburg, Austria; renata.delgiudice@asst-santipaolocarlo.it (R.d.G.); manuel.schabus@sbg.ac.at (M.S.); 2 Laboratory for Sleep, Cognition and Consciousness Research, University of Salzburg, 5020 Salzburg, Austria; 3 Department of Mental Health, University of Milan, 20142 Milan, Italy; 4 San Paolo University Hospital, ASST Santi Paolo e Carlo, 20142 Milan, Italy

**Keywords:** perinatal memory, fetal learning, quiet sleep, active sleep, fetus, newborn, maternal voice, EEG

## Abstract

In a pilot study, 34 fetuses were stimulated daily with a maternal spoken nursery rhyme from week 34 of gestation onward and re-exposed two and five weeks after birth to this familiar, as well as to an unfamiliar rhyme, both spoken with the maternal and an unfamiliar female voice. During auditory stimulation, newborns were continuously monitored with polysomnography using video-monitored *hd*EEG. Afterward, changes in sleep–wake-state proportions during familiar and unfamiliar voice stimulation were analyzed. Our preliminary results demonstrate a general calming effect of auditory stimulation exclusively in infants who were prenatally “familiarized” with a spoken nursery rhyme, as evidenced by less waking states, more time spent in quiet (deep) sleep, and lower heartrates. A stimulation naïve group, on the other hand, demonstrated no such effects. Stimulus-specific effects related to the familiarity of the prenatally replayed voice or rhyme were not evident in newborns. Together, these results suggest “fetal learning” at a basic level and point to a familiarization with auditory stimuli prior to birth, which is evident in the first weeks of life in behavioral states and heartrate physiology of the newborn.

## 1. Introduction

Infants’ sleeping habits are a constant concern for parents, including the question of why some infants can drift off to sleep so easily and still sleep on even in unfamiliar or noisy environments, whereas others simply cannot in the presence of the slightest auditory cues. Besides genetic and biological reasons, one plausible answer could be infants’ prenatal experiences, often also referred to as “fetal programming.” The individual variance that fetuses experience when it comes to auditory exposure depends on the everyday maternal environment and is strongly influenced by factors such as the mother’s daily routines and social habits or exposure to environmental noise.

As a pilot study for a planned longitudinal examination regarding the perinatal impact of stress on infants’ development, we explored the question of prenatal influences on newborns’ sleep and physiology. In detail, we were interested in whether repeated prenatal auditory stimulation with a taped maternal spoken nursery rhyme leads to familiarization in fetuses and therefore less sleep disruptions in auditory re-exposed newborns. Furthermore, we were interested in the specific effects of stimulus familiarity (maternal vs. unfamiliar voice; prenatally familiarized vs. unfamiliar nursery rhyme) on infant’s sleep–wake-states and physiology.

What is already known about sleep during the perinatal period? Third-trimester fetuses and newborns are known to spend the vast majority of their time sleeping [1,2], which is compatible with the particular relevance of sleep for brain and body development. As the circadian rhythm is not fully established even in healthy full-term newborns, newborns are known to show an irregular yet extensive pattern of sleep (up to 18 h) and wakefulness during day and night, with sleep often only interrupted by feeding periods [3]. Newborn’s sleep is divided into three stages [1]: Quiet sleep (QS; similar to adult non-rapid-eye-movement sleep) with no or minimal body and eye movements, regular respiration and heartrate; active sleep (AS; similar to adult rapid-eye-movement sleep) with irregular respiration, facial grimaces, smiling, sucking, muscle twitches, and gross limb movements; as well as transitional sleep (TS), which usually marks transitions from wake (W) to AS and vice versa. In contrast to older infants, the newborn sleep cycle usually starts with AS and is characterized by an equal proportion of AS and QS [3], with AS steadily decreasing for the sake of more QS over the first weeks of life [4].

Sleep and sleep patterns in newborns are influenced by several factors. Besides central nervous system maturation [5], the depth of sleep is reported to be influenced by the sleeping position (QS is enhanced in supine vs. prone position) [6], as well as caregiving practices (such as the amount of maternal skin-to-skin contact) [7]. Additionally, there are a few studies investigating changes in newborn’s sleep patterns in response to environmental noise. For example, QS is found to be increased in preterms after reducing ambient noise [8,9] and sleep disruptions increase to unfamiliar sounds in preterm infants, even if sound peaks were only moderately above background noise levels [10].

On the other hand, Filippa et al. [11] found that listening to the maternal voice—probably the most salient stimulus for newborns—leads to arousing shifts from sleep to wake when newborns were exposed to maternal speech but not when they were exposed to the singing voice of the mother [11]. It is noteworthy that, in past studies, the maternal voice is sometimes interpreted as arousing [12], whereas in other studies, it is interpreted as rather calming [13,14], as often evidenced by heartrate changes. Only a few of the studies published actually report the behavioral state (wake, AS, QS) of the babies at stimulation onset. Eventually, the differential behavioral reactions (calming vs. arousing) found in response to auditory stimulation may thus be related to such state effects.

Our study now focuses explicitly on sleep-wake-state changes to (prenatally presented or not presented) auditory stimuli. Given the literature, it is well documented that even newborns stay receptible to specific incoming information even at overt unconscious and “offline” periods of sleep. In detail, it has been reported that sleeping newborns are able to distinguish between various auditory stimuli, as demonstrated for speech vs. music [15], standard vs. deviant vowels [16,17], and forward vs. backward voices [18]. Furthermore, sleeping infants are reported to detect prosodic speech cues [19], discriminate anger and fear in voices [20], and even learn during sleep, as evidenced by increased eye movement responses to a tone earlier paired with an air puff [21,22]. Also, sleeping newborns (aged two days to six weeks) are reported to show heartrate de- or accelerations to various auditory stimuli [11,23,24]. However, there is an interesting individual variation in the intensity level needed to evoke a response in newborns during different behavioral states (for an overview see [24]). Additionally, it has been demonstrated that fetuses’ [25,26] and newborns’ [24,27,28] responses to sounds are, amongst others, dependent on previous sound exposure. Surprisingly, these responses to familiar stimuli are sometimes reported to be stronger [24,28], indicating an orienting response, and sometimes weaker [25,26,27], indicating habituation, as compared to new and unfamiliar stimuli.

These stimulus familiarity studies allow the investigation of the earliest forms of memory, as its origins lie in some kind of learning or “familiarization” before stimulus re-exposure. Evidence for auditory learning in fetuses (i.e., differential responses to familiar vs. unfamiliar stimuli) has been reported from the third trimester of gestation onwards. Fetuses are able to learn music [28,29], voices, stories, and nursery rhymes (for an overview, see Kisilevsky & Hains [30]), as evidenced by fetal heartrate changes to these familiar stimuli. Furthermore, decreasing startle responses or movements to a repeatedly presented vibroacoustic stimulus has been shown at this early stage [25,26], as well as habituation effects to repeatedly presented tones on a brain-level using fetal Magnetoencephalography (MEG) [31]. Additionally, behavioral state transitions have been reported to become more prevalent when fetuses are re-exposed to familiar music [28].

Such kinds of fetal memories have been shown to persist into the neonatal period and the first months of life. The most studied stimulus in this context is again the maternal voice. After birth, newborns are reported to react quite differently to the maternal voice, as evidenced by changes in sucking behavior of pacifiers [32,33], decreasing amount of movements [34], or fewer behavioral signs of pain during blood sampling when the maternal voice has been replayed at the same time [35].

Besides the maternal voice, there is also evidence that prenatally presented music is recognized at birth, as evidenced by heartrate (HR) decreases [36] and sleep to wake transitions in newborns [28]. In summary, there is good evidence for prenatal learning and the retention of prenatal memory traces up to the postnatal period.

In the present study, we therefore examined changes in behavioral states (W, QS, AS) to auditory stimulation in prenatally familiarized (EG) or stimulation naïve (CG) newborns. For that purpose, we continuously monitored newborns with polysomnography (i.e., using video-monitored high density electroencephalography; *hd*EEG), including electromyogram, electrooculogram, electrocardiography and videography) at week two and week five after birth in their home environment when they were exposed to prenatally presented “familiar” or new “unfamiliar” nursery rhymes in either the voice of the mother or an unfamiliar female voice. Despite mixed earlier findings, we hypothesized increased waking during stimulation with the unfamiliar rhyme in the prenatally familiarized EG. Furthermore, we hypothesized that the familiar (maternal) voice will calm all newborns (EG and CG) and result in less waking. Additionally, we expected in general less arousal, indicated by lower heartrates, in prenatally stimulated newborns (EG).

## 2. Materials and Methods

### 2.1. Participants

Forty-five pregnant women with a minimum age of 18 years and complication-free pregnancies were recruited from 2016 to 2018 at meetings for parents-to-be of local hospitals. An additional sample of ten participants dropped out before postnatal testing due to premature birth or pregnancy-related health problems. From the 45 babies included in the full study, 11 infants (EG = 6; CG = 5) had to be later excluded from data analysis due to uncorrectable artifacts (from movements, crying, and/or bad electrodes) in physiological measures and as a consequence ambiguous sleep staging scores. The final sample, therefore, included data from 34 infants (EG = 23; CG = 11) with a mean age of 14.78 days (*SD* = 4.29) in the first recording and a mean age of 36.65 days (*SD* = 4.49) in the second recording. All infants were born full-term (>38 weeks of gestation, mean = 39.45) and healthy. Mothers-to-be were on average 31.47 years (*SD* = 4.29) old and native German speakers. Fifty-three ercent were married, 47% were unmarried but living with their partner, and 42% had a university degree. Participants gave written informed consent at their first visit to our laboratory. The presented study was approved by the ethics committee of the University of Salzburg (EK-GZ 12/2013).

### 2.2. Materials

We used two different ecologically valid nursery rhymes for auditory stimulation, which were different in rhythm (calm vs. lively). Both rhymes were taped with the individual maternal voice and an unfamiliar female voice (a professional female speaker, identical over all recordings). Using the freely available software Audacity® (iWeb Media Ltd., Birkirkara, Malta [37]), we standardized the total length of each rhyme to exactly 60 s per rhyme. As twice daily (morning and evening) prenatal stimulation (with one rhyme) was planned to last five minutes per stimulation, a loop with five repetitions was created with the originally taped rhyme, resulting in five rhyme repetitions per stimulation day. For stimulation after birth, stimuli were randomly presented over speakers (60 dB) with a laptop using the presentation software Presentation (NeuroBehavioral Systems, Berkeley, CA, USA [38]).

### 2.3. Experimental Procedure

**Figure 1:**
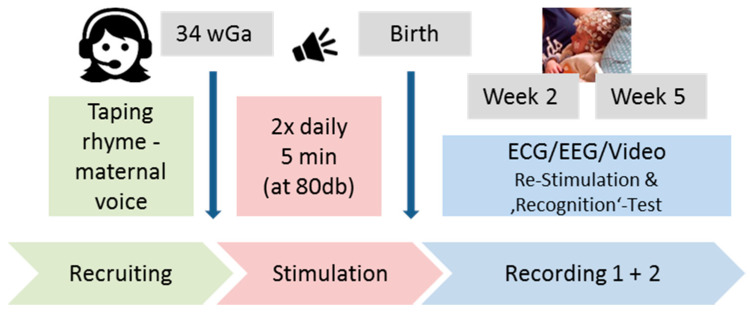
Experimental procedure. Pregnant women (*n* = 34) were asked to tape two different nursery rhymes and were afterward randomly assigned to two groups. The experimental group (EG, *n* = 23) replayed one rhyme (80 dB over speakers; twice a day for five minutes) from week 34 of gestation until birth (i.e., up to ~42 days). The control group (CG, *n* = 11) did not replay any rhyme. Two and five weeks after birth infants’ electrocardiography (ECG) and high density electroencephalography (*hd*EEG) were recorded during baseline (silence) and auditory stimulation periods with both rhymes, each presented with the maternal and an unfamiliar female voice.

The experimental protocol was divided in a prenatal and postnatal stimulation part (see Figure 1). In preparation for the prenatal stimulation, mothers-to-be visited our laboratory before gestational week 34 and taped the two different nursery rhymes mentioned before. The experimental group (*n* = 23) received one of the two maternal spoken rhymes (i.e., randomly chosen rhymes of different rhythm) taped on a CD and replayed it over speakers (80 dB) from gestational week 34 on until birth, as we expect 20 dB dampening across the mothers’ belly according to Gerhardt and Abrams [39]. Auditory stimulation took place twice a day (morning and evening) for five minutes (five repetitions of the 60 s lasting nursery rhyme) in the home environment of mother and child. Mothers in the EG were instructed to sit and relax in a quiet room and avoid touching their belly during auditory stimulation. Daily stimulation frequency, stimulation sound pressure level, and mothers’ well-being were verified and documented using a tablet at the mothers’ homes. An additional control group (*n* = 11) of mothers did not play any rhyme before birth but completed an identical study protocol thereafter.

At about two and five weeks after birth, mothers and infants were visited in their home environment (except for four mothers, who decided to come to the lab for postnatal recordings). After instructing the participants, an *hd*EEG cap [40] was placed on the infant’s head, and a camera, speakers, and laptop for stimulus presentation were set up. Polysomnography (PSG) including infants’ electrocardiography (ECG), *hd*EEG, and video were collected during baseline (silence) periods as well as during auditory stimulation with the two different nursery rhymes, taped and spoken by the mother (familiar voice) and by a professional female speaker (unfamiliar voice). Recording time was in total 27 min, with three minutes (= three repetitions of the one-minute rhyme) for each of the four randomized presented stimulus condition (2 rhymes × 2 voices) and a three-minute baseline (silence) before each (and after the last) stimulus. Within about five weeks after birth, the same experimental setting was repeated. The experimental setup after birth was identical for the experimental and control group.

### 2.4. Electrophysiological Data Collection

For EEG acquisition, a 128 electrodes GSN HydroCel Geodesic Sensor Net (Electrical Geodesic Inc., Eugene, OR, USA [40]), provided in three different sizes (34–36, 36–37, 37–38 cm), and a Net Amps 400 amplifier (Electrical Geodesic Inc., Eugene, OR, USA [40]), were used. Electromyogram (EMG) and horizontal electrooculography (EOG) were collected using electrodes from the EEG net, for additional vertical EOG, two additional electrodes were placed above and below the infant’s left eye. Two further electrodes were placed diagonally above the infant’s right clavicle and left abdomen for ECG measurements. EEG and ECG data were recorded with a sampling rate of 1000 Hz. During electrophysiological data collection, all babies were lying in the arm of their mothers, who were instructed to keep their baby still or, if the baby gets uneasy, make only gentle rocking movements.

### 2.5. Sleep Staging Analysis

Sleep staging was based on six EEG (F3, F4, C3, C4, O1, O2), bipolar ECG/EMG/VEOG, and HEOG channels (i.e., using a classical PSG setup). Every 30-sec-epoch was assigned to wake (W), active sleep (AS), quiet sleep (QS), transitional sleep (TS), or artifact-rich epochs of movement (X), according to Scholle and Feldmann-Ulrich [41]. Simultaneously, videos taped during data acquisition were also analyzed in 30-sec-epochs, according to Prechtl States 1–5 [42]. Afterward, sleep staging and Prechtl states were compared by a second rater, and in case of a mismatch (e.g., AS in PSG vs. wake in Prechtl), sleep staging results were corrected. In the final analysis, only subjects with unambiguous sleep staging scores were included, and epochs with transitional sleep and movement artifacts were excluded from analysis.

### 2.6. ECG Preprocessing and Analysis

ECG data was preprocessed using Matlab (The MathWorks, Inc., Natick, MA, USA [43]) software with the Anslab Professional [44] toolbox. After correcting for artifacts and incorrectly detected R-peaks, the data was—corresponding to sleep staging analysis—segmented in 30 s epochs, and infants’ mean heartrate was calculated for every epoch.

### 2.7. Statistical Analyses

Statistical analyses were performed using IBM SPSS Statistics 24 [45] and based on repeated measures and mixed analyses of variance (ANOVA). Significance levels were set to *p* < 0.05 and effect sizes are reported as partial eta squared (p.eta^2^) and Cohen’s d (d). Greenhouse-Geisser correction was utilized for violations of sphericity.

To test whether auditory stimulation influences the sleep–wake cycle, the time (in seconds) spent in W, AS, and QS was calculated for every subject. Afterward, we calculated the mean time spent in W, AS and QS for four baselines (180 s each; or six behavioral state values with 30 s duration) and four stimulation phases (also 180 s each). The effect of voice familiarity on sleep–wake states was computed by further dividing previously mentioned stimulation periods into familiar voice and unfamiliar voice. To examine the effect of rhyme familiarity in the EG, we repeated the described procedure and divided stimulation periods into familiar and unfamiliar rhyme. To examine whether auditory stimulation affects the autonomic nervous system reaction (measured by heartrate), the sleep staging data and the corresponding mean heartrates were matched for every 30-second epoch and divided into baseline and stimulation periods. Afterward, mean heartrates for AS, QS, and W during baselines and stimulation periods were calculated for every subject.

## 3. Results

### 3.1. Effect of Auditory Stimulation on Sleep-Wake-States

Repeated measures ANOVA with the independent variables and within-factors behavioral STATE (QS, AS, W), PHASE (baseline, stimulation) and AGE (2 weeks, 5 weeks) and the between-subject factor GROUP (prenatal stimulation/experimental group = EG, no prenatal stimulation/control group = CG) were computed. The dependent variable was the time (in seconds) spent within a certain behavioral state (W, AS, or QS). Importantly, the amount of overall staged sleep epochs is thereby not different between baseline and simulation periods at both recordings two (*t*(33) = −1.56, *p* = 0.129) and five (*t*(33) = −0.749, *p* = 0.459) weeks after birth. Further analyses revealed that, overall, the time spent in the three behavioral states was different for W, QS, and AS (*F*(1.53, 49.03) = 14.71, *p* < 0.001, *p.eta^2^* = 0.32), with most of the time spent in active sleep, followed by wake and quiet sleep (for details please refer to suppl. materials). In addition, the time spent in the three behavioral states tended to be different between the EG compared to the CG, as indicated by the interaction STATE*GROUP (*F*(2, 64) = 2.82, *p* = 0.067, *p.eta^2^* = 0.08). Exploratory post-hoc independent t-test revealed (*t*(32) = −1.89, *p* = 0.068) that the time spent in AS tended to be higher in the EG group (*M* = 421.60, *SE* = 32.10) as compared to the CG (*M* = 302.05, *SE* = 62.48) and also the time spent in W tended overall to be lower in the EG (*t*(32) = 1.73, *p* = 0.093; EG: *M* = 143.15, *SE* = 32.24 vs. CG: *M* = 256.36, *SE* = 67.08).

**Figure 2:**
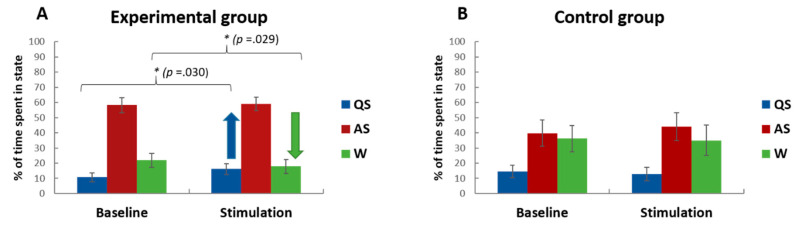
Behavioral states in response to auditory stimulation in infants prenatally exposed to (experimental group, EG) and unexposed to (control group, CG) auditory stimulation. Figures show the proportions of time spent in quiet sleep (QS), active sleep (AS), and wake (W) states during (4 × 3 min) baseline (silence) and during (4 × 3 min) auditory stimulation conditions in infants who were prenatally exposed to auditory stimulation (**A**; EG; *n* = 23) and who were not exposed (**B**; CG; *n* = 11). Note that infants familiar to prenatal stimulation (EG; **A**) fell asleep more likely (less W) as well as slept deeper (more QS) during auditory stimulation and generally exhibited less W states at baseline and stimulation. On the other hand, unexposed infants (CG, **B**) exhibit a pattern indicating no changes in behavioral states from baseline to stimulation. Curly brackets highlight the significant post-hoc t-test comparisons. * = statistical significance *p* < 0.05; Error bars refers to ±1 Standard Error of the Mean (SEM).

The interaction STATE*PHASE indicates another trend for the EG (*F*(2, 44) = 2.89, *p* = 0.066), and no such effect for the CG (*F*(2, 20) = 1.17, *p* = 0.331, *p.eta^2^* = 0.10) when focusing on the change from baseline to stimulation for the three behavioral states of interest (cf. Figure 2). Exploratory post-hoc paired t-tests thereby revealed that in the EG group the time spent in W decreased (*t*(22) = 2.32, *p* = 0.029) from baseline (*M* = 157.82, *SE* = 33.53) to stimulation periods (*M* = 154.22, *SE* = 32.16), whereas QS increased (*t*(22) = −2.32, *p* = 0.030) from baseline (*M* = 77.61 s, *SE* = 21.12 s) to stimulation periods (*M* = 116.73, *SE* = 25.70). Non-parametric bootstrapping analysis (1000 repetitions) confirms that W is reduced in the EG from baseline to stimulation (t(22) = 2.32, *p* = 0.033), whereas QS is increased from baseline to stimulation t(22) = −2.32, *p* = 0.044) which we interpret as calming effect of the stimulation in the (auditory stimulation) familiarized EG.

### 3.2. Effect of Voice Familiarity on Sleep-Wake-States

Repeated measures ANOVA with the within-subject factors behavioral STATE (QS, AS, W), PHASE (baseline, stimulation), VOICE (familiar, unfamiliar), and AGE (2 weeks, 5 weeks) revealed a main effect for STATE (*F*(1.48, 48.91) = 20.47, *p* < 0.001, *p.eta^2^* = 0.38), but no interaction for STATE*VOICE (*F*(1.58, 51.30) = 1.71, *p* = 0.196, *p.eta^2^* = 0.05) or STATE*PHASE*VOICE (*F*(1.35, 44.58) = 1.60, *p* = 0.215, *p.eta^2^* = 0.05; cf. Supplemental Figure S1), indicating that voice familiarity did not influence the amount of time spent in the three different behavioral states.

### 3.3. Effect of Rhyme Familiarity on Sleep–Wake-States

In addition to the effect of voice familiarity, we also tested whether rhyme familiarity due to prenatal stimulation was evident at two and five weeks after birth. Rhyme familiarity was tested using a repeated-measures ANOVA with the within-subject factors behavioral STATE (QS, AS, W), PHASE (baseline, stimulation), RHYME (familiar, unfamiliar), and AGE (2 weeks, 5 weeks) during stimulation in the experimental group (EG). Analysis revealed that the time (in seconds) overall spent in the three behavioral STATEs was different (*F*(1.39, 30.59) = 35.23, *p* < 0.001, *p.eta^2^* = 0.62) with more time spent in AS. However, the interaction behavioral STATE*RHYME familiarity (*F*(1.44, 31.70) = 0.21, *p* = 0.752, *p.eta^2^* = 0.01; cf. Supplemental Figure S2) was not significant, indicating that time spent in behavioral states did not change in response to familiar vs. unfamiliar rhyme exposure after birth. Likewise, the time spent in the three behavioral STATES did not change as a function of RHYME familiarity from baselines to stimulation (PHASE*RHYME*STATE: *F*(2, 44) = 0.24, *p* = 0.587, *p.eta^2^* = 0.02).

### 3.4. Effect of Auditory Stimulation on Physiology

**Figure 3:**
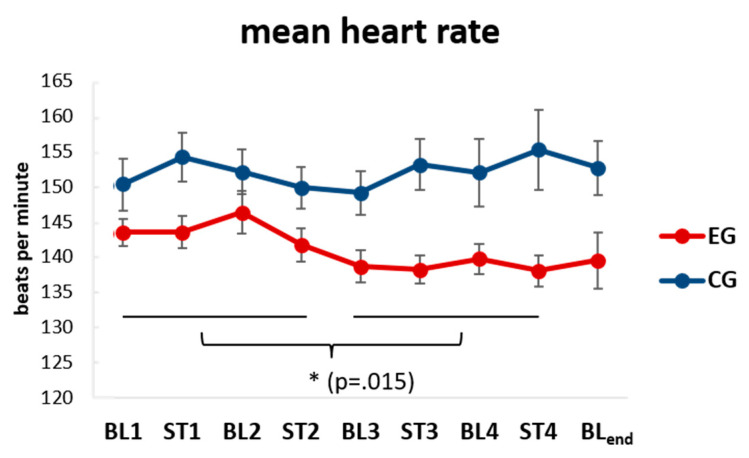
Evolution of the heartrate (HR) over time. The two timelines show the mean HR for the four chronologically ordered baseline (BL_1,2,3,4_) and stimulation (ST_1,2,3,4_) periods of 3 min each independent of rhyme and voice familiarity. Note that infants familiar (EG) and unfamiliar (CG) with prenatal stimulation show distinct HR. Especially in the second part the EG seems to show habituation and deceleration of the HR as revealed by paired-sample t-tests. Data is pooled from the same babies recorded twice at week two and week five of age. BL_end_ refers to the last 3 min baseline following four baseline and stimulation periods. * = statistical significance *p* < 0.05; Error bars = ±1 SEM.

In addition to the analysis of behavioral state shifts in response to the re-exposure with the familiar and unfamiliar auditory stimuli, we complemented the picture with analyses of babies’ physiology, namely heartrate (HR) irrespective of rhyme and voice familiarity. Analysis of HR using a three-way ANOVA with TIME (9 × 3 min periods), AGE (2 weeks, 5 weeks), and GROUP (EG, CG) revealed a main effect for GROUP (*F*(1, 29) = 8.99, *p* = 0.006, *p.eta^2^* = 0.24). Post-hoc independent t-tests indicate a lower mean HR in the EG (*M* = 140.66, *SD* = 7.81) as compared to the CG (*M =* 152.56, *SD* = 10.71) during baseline and stimulation periods. Additionally, comparing the first half (12 min; BL1-ST2) and the second half (12 min; BL3-ST4) of the recordings revealed that the EG, but not the CG, reduced HR (*t*(20)= 2.67, *p* = 0.015, *d* = 0.57) from the first half (*M* = 143.71, *SD* = 8.99) to the second half (*M* = 138.64, *SD* = 8.83) of the recording (cf. Figure 3). Data, therefore, indicates habituation over the course of the recording exclusively for the prenatally familiarized EG but not the (auditory stimulation-) naïve and unexposed CG. For more details, see Supplemental Table S1.

## 4. Discussion

The present study aimed to investigate whether the effects of repeated auditory stimulation during pregnancy are visible in the first weeks after birth. Specifically, we hypothesized that daily prenatal exposure to a nursery rhyme would lead to “recognition,” and therefore habituation, to that rhyme two and five weeks after birth, as evidenced by more calm and relaxed newborns during stimulation with the familiar nursery rhyme. Interestingly, we found no stable effect of stimulus familiarity on the newborns’ sleep-wake states, neither for rhyme, nor voice familiarity in the prenatally exposed experimental group. However, what was found was a general effect indicative of “fetal programming.” We observed that only those babies who were already prenatally exposed and familiarized to an auditory nursery rhyme on a daily basis (experimental group, EG) calmed down when these rhymes (regardless of familiarity) were replayed after birth, whereas the prenatally unstimulated naïve control group showed no such effects. The former finding in the EG was also replicated in newborns’ physiological responses with a progressive slowing down of the heartrate across the recording period for the pre-exposed EG only. A similar calming effect during re-exposure with auditory stimulation was reported earlier by Granier-Deferre et al. [36], who found HR decelerations in infants re-exposed to a familiar (prenatally presented), but also to an unfamiliar, piano melody. Daily prenatal exposure to auditory nursery rhymes seems to have familiarized babies to such environments after birth as the unexposed CG showed a higher percentage of wake states during exposure with the nursery rhymes as well as a generally elevated heartrate. It is to note that this is not due to awakenings during auditory stimulation in the CG, but EG infants (familiarized prenatally with auditory stimulation) generally seemed calmer and sleepier during auditory (re-) exposure. This is in line with findings from Gonzalez-Gonzalez et al. [27], who reported that newborns repeatedly exposed to a (vibroacoustic) stimulus in utero habituated earlier to the same stimulus after birth. Muenssinger et al. [31] have also reported habituation to repeatedly presented auditory stimulation even in third-term fetuses using fetal MEG, suggesting that the neuronal basis for learning is already present before birth. Another less likely explanation for the observed calming effect could be, that newborns are linking the re-exposure to the nursery rhymes to the prenatal auditory stimulation setting, where mothers-to-be were instructed to sit calmly and relax while listening to the rhymes via loudspeakers. It is possible that this immobile relaxed setting was linked to the auditory stimulation and results in more relaxed fetuses and, therefore, more relaxed newborns at auditory re-exposure after birth. Altogether, daily auditory stimulation of our third-trimester fetuses may, therefore, have resulted in basic memory for, and quicker habituation to, such conditions. Infants not being familiarized before birth, on the other hand, may react with irritation or attention orientation to environmental (speech-like) noise. The most interesting point here is that prenatally familiarized infants calmed down regardless of stimulus familiarity and, additionally, despite the auditory stimulus being significantly distorted by, e.g., the amniotic fluid during the pre-natal stimulation period in utero. This finding indicates generalization of prenatally learned material in newborn infants.

However, whether prenatal learning is possible despite or due to the special significance of sleep for memory formation during the perinatal period is still a question to be answered. Some recent studies have shown that memory in young infants benefits from [46] and generalizes during sleep [47] as early as three months after birth. Whether perinatal sleep just before or after birth has similar significance is yet unknown but would be highly interesting as possibly stimuli presented at two weeks of life in our study may already be learned and recognized as “familiar” at our five-week follow-up.

Contrary to earlier studies showing stable calming effects for the maternal voice [13,14,35], we found no difference in the time spent in wake or sleep in the presence of the familiar maternal voice compared to periods when the unfamiliar female voice was present. As mentioned in the introduction, the maternal voice is sometimes also reported to be arousing for newborns [11,12]. As infants were in different behavioral states at stimulus onset, and therefore more (awake) or less (sleep) responsive to the stimulation with the maternal voice, we conclude this might be a potential reason for the missing effect in our study sample. Additionally, and also contrary to an earlier study [33], we found no distinct changes in the time spent in behavioral states (W, AS, or QS) to the prenatally presented nursery rhyme. Given our current analysis, we conclude that memory of the prenatally replayed nursery rhyme is only encoded to a basic degree, which is not identifiable at a behavioral (i.e., sleep-wake stage transition) level. Further analysis on brain (EEG) and autonomic physiology (ECG) level) is necessary to see if such effects are evident at a more fine-grained physiological level.

As a limitation of our study, one could discuss the rather short duration of our recordings (~27 min) and therefore a limitation of segments available for each and every sleep stage per newborn. However, in our experience, this problem is hard to circumvent in a sample of healthy newborns in their habitual home environment. First, it seems impossible to plan the recordings in such a way that the infants are awake and attentive at the start of the experimental paradigms as newborns as young as two to five weeks do not yet exhibit regular sleep–wake-cycles and constantly fall asleep irrespective of time of day or environmental stimulation. In addition, already the preparation of the EEG-system, the ECG montage and setting up of the videography in the babies’ home takes time and appears tiring for the newborns. Second, we already used comfortable *hd*EEG-nets with the main aim to save valuable time in EEG setup and still observed that newborns often got uneasy and whiny after about 30 min (an earlier abandoned version of the paradigm lasted 45 min). Experimentally the 27 min registration, therefore, appeared as an optimal trade-off between calm and comfortable infants and the minimum amount of data needed for sensible ECG and EEG analysis. A second limitation is the sample size, and in particular, the unbalanced group sizes for experimental and control groups. As data is difficult to gather at this very early stage of life in healthy newborns (here, we already recorded 45 babies twice in their home environment using full polysomnography setups), we believe that this is the best that be done within a comprehensive pilot study. Finally, we can only speculate why the EG (familiarized to the daily stimulation) starts with a lower heartrate (Figure 3) already in the baseline. We believe that the EG may simply be less aroused by the environmental noise during the preparation in their home environment as they may be generally less “environmental noise sensitive” after their daily exposure to the loud (80 dB) rhyme stimulation before birth over the course of six weeks.

In conclusion, our preliminary data indicates how prenatal experience or “fetal programming” may have an effect on behavioral (sleep/wake states) and physiological (heartrate) reactions just weeks after birth as evident in less waking periods and HR habituation to stimuli heard already in the last trimester before birth. Parents and societies should be aware of such effects and may consider this in their parenting methods even before birth. This is not to say that we are in favor of overambitious stimulation of the fetus in order to maximize learning even before birth. Rather we call into attention that much of what a fetus is exposed to before birth—whether it is parental movements, touch, music, or speech—may shape the infants’ physiology and later perception of the world. In which exact way will be work for further generations of developmental scientists.

## Supplementary Materials

The following are available online at https://www.mdpi.com/2076-3425/10/8/511/s1, Figure S1. Effect of voice familiarity; Figure S2. Effect of rhyme familiarity; Table S1. Mean heartrates in experimental and control group; Audiofiles: Kindchen_calm.wav, Butzemann_lively.wav.
