# Supplementary material for: Sleep, Little Baby: The Calming Effects of Prenatal Speech Exposure on Newborns’ Sleep and Heartrate
Source: Brain Sci. 2020 Aug 2;10(8):511. doi: 10.3390/brainsci10080511 (PMC7464711; doi:10.3390/brainsci10080511)
Supplement: Supplementary file 1 [file brainsci-10-00511-s001.zip › brainsci-863718_supplements/brainsci-863718_supplements.docx]

Sleep, little Baby: Calming Effects of Prenatal Speech Exposure on Newborns’ Sleep and Heart Rate

Lang, A. ^1,2^, delGiudice, R. ^1^, & Schabus, M. ^1,2^

Affiliations:

1. Department of Psychology, University of Salzburg, Austria
2. Laboratory for Sleep, Cognition and Consciousness Research, Centre for Cognitive Neuroscience [CCNS], University of Salzburg, Salzburg, Austria

# **Supplemental material**

## *Descriptive statistics of sleep-wake-states*

After removing epochs including transitional sleep or movement-related artifacts (crying / dysregulated state), analyses of prenatally stimulated infants’ (the experimental group) sleep data revealed a mean of 11.59% (*SD=*19.80) QS, 57.88% (*SD=*27.27) AS and 22.28% (*SD=*27.15) W at the age of two weeks and 15.40% (*SD=*15.70) QS, 59.24% (*SD=*26.31) AS and 17.48% (*SD=*26.14) W at the age of five weeks. In infants unfamiliar with prenatal stimulation (the control group) analyses revealed a distribution of 5.50% (*SD=*12.05) QS, 46.40% (*SD=*36.76) AS and 38.45% (*SD=*35.14) W states at the age of two weeks and 21.97% (*SD=*22.23) QS, 37.50% (*SD=*30.70) AS and 32,77% (*SD=*34.80) W with five weeks.
Independent t-test revealed trends regarding the differences in the time spent in W (*t*(32)= 1.73, *p* = 0.93) between experimental (*M=*19.88%*, SE=*4.48) and control groups (*M=*35.61%, *SE=*9.32*),* as well as in the time spent in AS (*t*(32)= -1.89, *p* = 0.68) between experimental (*M=*58.56%*, SE=*4.45) and control groups (*M=*41.96%, *SE=*8.68*)* pooled over both recordings.

## *Effect of voice familiarity on sleep-wake-states*

C

The time (in %) spent in different behavioral states (QS, AS, W) during the presentation of the familiar (= maternal) voice, and the unfamiliar (= unknown female) voice.


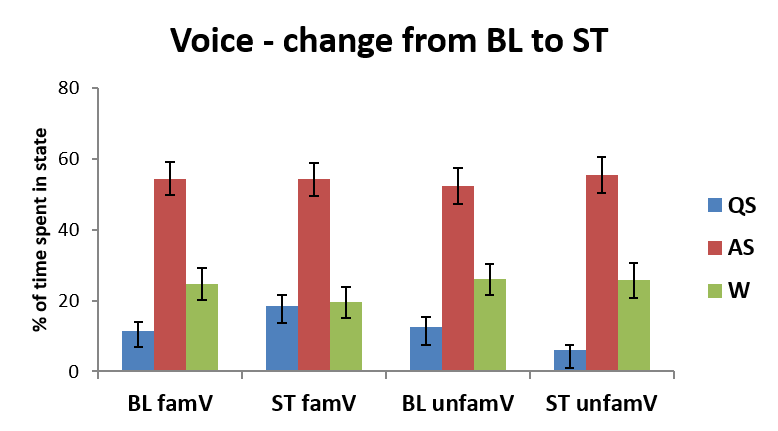


*Figure S1*. **Effect of voice familiarity.** Voice familiarity did not induce a significant change in the time spent in behavioral states (QS, AS, W) during baseline and stimulation periods. Data is pooled from the same babies recorded twice at week 2 and week 5 of age. QS = quiet sleep, AS = active sleep, W = wake, BL = baseline, ST = stimulation, famV = familiar voice, unfamV = unfamiliar voice; Error bars = +/- 1 SEM.

*Effect of rhyme familiarity on sleep-wake-states*

The time (in %) spent in different behavioral states (QS, AS, W) during the presentation of the familiar (= prenatal presented) rhyme, and the unfamiliar (= not presented control) rhyme.


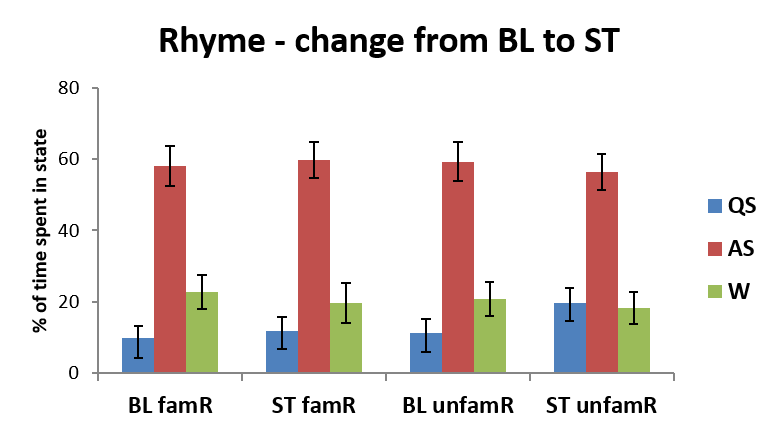


*Figure S2*. **Effect of rhyme familiarity.** The EG who was exposed to one of two rhymes prenatally does not show a significantly different proportion of sleep-wake states at re-exposure with the familiar vs. an unfamiliar rhyme after birth. Data is pooled from the same infants recorded twice at week 2 and week 5 of age. QS = quiet sleep, AS = active sleep, W = wake, BL = baseline, ST = stimulation, famR = familiar rhyme, unfamR = unfamiliar rhyme; Error bars = +/- 1 SEM. .

## *Table S1. Mean heart rates in experimental and control group*

Descriptive statistics of mean heart rates at both recordings. Significant (p<.05) effects highlighted in bold and marked with asterisk.


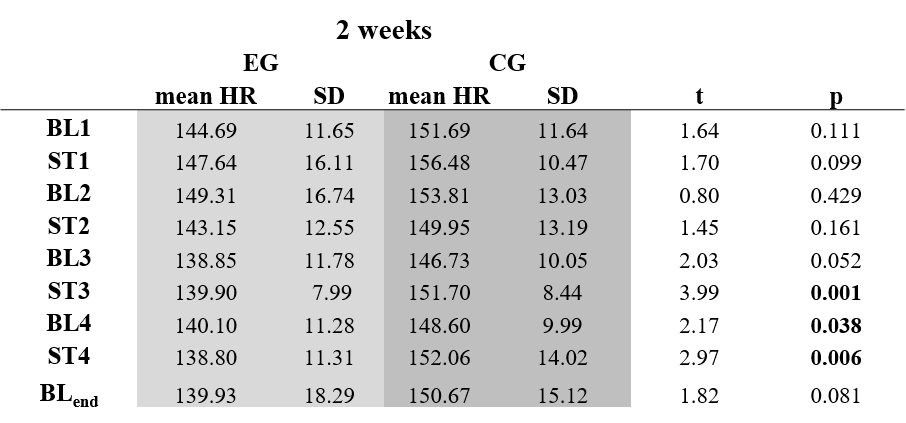


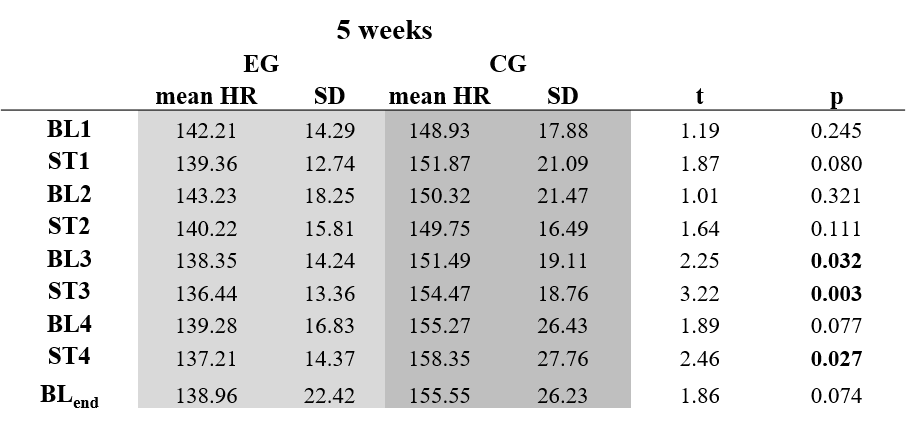


Note. The tables show newborns mean heart rates in chronologically ordered and randomly presented resting (baseline, BL) and stimulation (ST) periods and comparisons for all periods between infants familiar (EG) and unfamiliar (CG) with prenatal stimulation. Groups show significant differences in the second half of the recordings, marked in bold font. EG=experimental group; CG=control group
